# A novel *SEPT12* mutation, T96I, is associated with sperm head and annulus defects

**DOI:** 10.3389/fcell.2024.1498013

**Published:** 2025-01-07

**Authors:** Kuan-Ru Chen, Han-Yu Wang, Yung-Che Kuo, Yu-Chih Lo, Pao-Lin Kuo

**Affiliations:** ^1^ Department of Medical Research, E-Da Hospital, I Shou University, Kaohsiung, Taiwan; ^2^ Department of Obstetrics and Gynecology, Jen-Ai Hospital, Taichung, Taiwan; ^3^ Department of Obstetrics and Gynecology, Kaohsiung Chang Gung Memorial Hospital and Chang Gung University College of Medicine, Kaohsiung, Taiwan; ^4^ TMU Research Center for Cell Therapy and Regeneration Medicine, Taipei Medical University, Taipei, Taiwan; ^5^ Department of Biotechnology and Bioindustry Sciences, College of Bioscience and Biotechnology, National Cheng Kung University, Tainan, Taiwan; ^6^ Department of Obstetrics and Gynecology, National Cheng Kung University Hospital, National Cheng Kung University, Tainan, Taiwan

**Keywords:** SEPTIN12, sperm head, sperm annulus, infertility, mutation

## Abstract

Infertility affects around 8%–12% of reproductive-aged couples and is a major health concern. Both genetic and environmental factors influence male infertility. *SEPTIN12* is a crucial testis-specific gene essential for the final differentiation of male germ cells and is strongly linked to male infertility due to numerous detected mutations. The present study identified a novel *SEPTIN12* ^T96I^ mutation that causes male infertility. Immunofluorescence staining and transmission electron microscopy (TEM) analysis of T96I sperm revealed co-localization of SEPT12 and SEPT7 in the obliquely positioned annulus. In addition, the sperm carrying the T96I mutation demonstrated large nuclear vacuoles, irregular swelling, and decondensation of the acrosomal cap. The overexpression of SEPT12 T96I in NT2/D1 cells impaired the formation of SEPT7 filaments, emphasizing the significance of SEPT12 filaments for sperm morphology and function. Our results demonstrate the importance of *SEPTIN12* ^T96I^ in male infertility and offer valuable insights for future detection in infertile men.

## 1 Introduction

Infertility affects about 8%–12% of reproductive-aged couples worldwide and is recognized as a major health concern (Vander Borght and Wyns, 2018). Male infertility can present with a wide range of conditions, from normal semen parameters to a complete absence of sperm production. It includes low sperm concentration, poor sperm motility, or abnormal morphology (Vander Borght and Wyns, 2018). Male infertility is multifactorial, involving both genetic and environmental factors (Agarwal et al., 2021). Genetic variations significantly contribute to male infertility, highlighting the urgent need for further exploration into the genetic basis of sperm head and annulus defects (Houston et al., 2021). Therefore, understanding the genes responsible for male infertility and their mechanisms is essential for diagnostics and our comprehension of the condition.

Septin (SEPT) family proteins are a family of conserved cytoskeletal proteins found from yeast to mammals (Benoit et al., 2023). They have a highly conserved GTP-binding domain with GTPase activity and play roles in various developmental and physiological processes (Lin et al., 2019). *SEPTIN12* is a pivotal testis-specific gene essential for the terminal differentiation of male germ cells (Lin et al., 2009). Numerous mutations of *SEPTIN12* have been detected in infertile males, strongly indicating that *SEPTIN12* is a pivotal gene linked to male infertility (Kuo et al., 2012; Li et al., 2021; Quarantani et al., 2023; Rafaee et al., 2020). *SEPTIN12* mutations are suggested to be associated with acrosome, head, neck, and annulus defects, and decreased mobility (Kuo et al., 2012; Li et al., 2021; Lin et al., 2009). In mouse model, *Sept12* ^+/−^, *Sept12* ^−/−^ and *Sept12* ^*D197N/D197N*^ mouse spermatozoa present significant defects, such as round spermatids, headless, bent necks, acrosome, and tail defects, and decreased mobility (Chen et al., 2022; Lin et al., 2009; Shen et al., 2020).

The SEPT12 protein plays a crucial role in sperm head-tail formation and is a vital component of the sperm tail annulus (Lin et al., 2019). The annulus, located between the midpiece and principal piece of mammalian sperm flagellum, prevents mitochondrial diffusion and displacement (Caudron and Barral, 2009). The sperm annulus consists of SEPT12 and SEPT7, SEPT6, and SEPT4, which form octameric filaments that polymerize end-to-end (Kuo et al., 2015). The GTP-binding domain of SEPT12 plays a vital role in facilitating its interaction with SEPT7 (Kuo et al., 2015). The phosphorylation of SEPT12 impairs filament formation, ultimately resulting in abnormal sperm structures and a decline in male fertility (Shen et al., 2017). In addition, the phosphorylation of SEPT12 and SEPT4 activity is crucial for sperm capacitation (Wang et al., 2023). Identifying novel mutations in SEPT12 may provide novel insights into the role of these SEPTs in the cell biology aspect.

Herein, we have discovered novel mutations T96I in the GTPase domain of SEPTIN12 that have not been explored in previous studies. The variant was found in the Genome Aggregation Database with an allele frequency of 8.68 × 10^−6^ and was reported as having uncertain significance. Two infertile patients with a novel dominant mutation in exon (c.287T > C, [p.T96I]) in *SEPTIN12*. *SEPTIN12* ^T96I^ sperm showed large nuclear vacuoles, irregular swelling, and decondensation of the acrosomal cap. Functional experiments have conclusively demonstrated the critical role of the T96 residue of SEPT12 in forming SEPT12-SEPT7 filaments. Our study found that *SEPTIN12* ^T96I^ is involved in sperm head and annuls development in humans, expanding our understanding of male infertility.

## 2 Materials and methods

### 2.1 Clinical information

The study, approved by the Institutional Review Board of National Cheng Kung University Hospital, enrolled 400 infertile men with abnormal semen parameters and 360 fertile men with normal semen parameters from January 2005 to July 2007 (Kuo et al., 2012). The infertile men had at least one of the following parameters: sperm concentration <20 x10^6^/mL, motile sperm <50%, or sperm with normal morphology <14% based on strict Kruger criteria. All patients underwent a comprehensive examination including history, physical examination, hormone profiling, and a molecular test for Y-chromosome microdeletions. Control subjects were husbands of women receiving prenatal care at the hospital, who had fathered at least 1 child within 2 years without assisted reproductive technologies and had normal semen parameters.

### 2.2 Mutation analysis

Genomic DNA was analyzed according to methods described previously (Kuo et al., 2012). In brief, genomic DNA was extracted from lymphocytes or saliva using a Puregene DNA isolation kit (Gentra, MN, United States). Specific primers were designed to amplify each coding region of the human *SEPTIN12* gene (GenBank accession no. NM_144605.3). The PCR products were directly sequenced with the same primer sets.

### 2.3 ClustalW multiple sequence alignment

The sequences of human SEPT12 orthologs from different species and various septins were aligned using the ClustalW2 program provided by EMBL-EBI.

### 2.4 Immunofluorescence staining

Human sperm were fixed with 4% paraformaldehyde in phosphate buffered saline (PBS). Cells and sperm were permeabilized with 0.1% Triton X-100 in PBS and blocked with the antibody diluent (Dako). Spermatozoa and cells were stained with anti-sept12 (H00124404-B01P, Abnova, Taipei, Taiwan), anti-sept7 (13818-1-AP, Proteintech, Rosemont, IL) or anti-FLAG (MilliporeSigma) antibody. After which, the cells were washed with PBS three times and were then incubated with secondary antibodies (catalog. A-11001, A-11011 or A-11004; Invitrogen) at room temperature for 1 h at room temperature. The cells were then further washed with PBS three times. The coverslips were overlaid on DAPI Fluoromount-G (Southern Biotech, Birmingham, AL) and the signals were detected through a fluorescence microscope (Olympus, Tokyo, Japan). For MitoTracker (Invitrogen) staining, cells were incubated with the dye for 15–30 min in PBS before fixation.

### 2.5 Transmission electron microscopy

Semen was collected through masturbation and washed three times with PBS. The sperm were then fixed in suspension with 2.5% glutaraldehyde at 4°C overnight. After the supernatant was removed, the sperm pellet was postfixed in 1% OsO₄ at room temperature for 1 h and subsequently washed with ddH₂O. The sperm were then dehydrated and prepared according to standard procedures for transmission electron microscopy. The samples were examined using a JEM-1400 transmission electron microscope.

### 2.6 Model building for the SEPT7-SEPT12 complex

The model of the SEPT7-SEPT12 complex was constructed by integrating the crystal structure of SEPT12 (PDB ID: 6MQB) with the cryo-EM model of the SEPT7-SEPT9 complex (PDB ID: 9BHW), both of which were obtained from the RCSB Protein Data Bank (https://www.rcsb.org/structure). SEPT12 and SEPT9, sharing a sequence identity of 62% and both belonging to the SEPT3 family, exhibit high structural similarity, with a root-mean-square deviation (r.m.s.d.) of 1.52 Å over 221 amino acids when superimposed using PyMOL.^2^ To construct the SEPT7-SEPT12 complex model, SEPT9 in the SEPT7-SEPT9 complex was substituted with SEPT12.

### 2.7 Plasmids construction and transfection

The generation of all *SEPTIN* constructs has been previously described. The full-length *SEPTIN12* cDNA was amplified by PCR and cloned into the pEGFP-N1 vector. The *SEPTIN12* ^T96I^ mutant construct using the QuikChange II site-directed mutagenesis kit (Stratagene, La Jolla, CA). Specific plasmids were transfected into NTERA-2 cl.D1 (NT2/D1) cells using Lipofectamine™ 2000 Reagent (11668500, Invitrogen, Carlsbad, CA) at 37°C for 5 h for transient transfection. After 18 h, the cells were processed for immunofluorescence staining or immunoblotting.

### 2.8 Immunoprecipitation and Western blotting

Cells were lysed with 1× RIPA buffer (10×, 20–188; Millipore, Temecula, CA) containing protease inhibitor for 10 min on ice. The lysates were incubated Dynabead-Antibody complex (Invitrogen, Vilnius, Lithuania) and rotated overnight at 4°C. Beads were collected by a brief centrifugation and washed three times with wash buffer (100 mM Tris, 150 mM NaCl, 2 mM ethylenediaminetetraacetic acid, 0.5% Tween-20% and 0.01% NP-40). Dynabead-Ab-Ag complexes were denatured by SDS sample buffer (6x sample buffer contains 360 mM Tris-HCl, pH 6.8, 12% SDS, 30% glycerol, 300 mM DTT) at 95°C 10 min. The samples were separated by 10% sodium dodecyl sulfate-polyacrylamide gel electrophoresis and transferred to PVDF membranes. The membrane was blocked with 3% BSA (Gibco, Auckland, New Zealand) dissolved in TBST at room temperature for 1 h and incubated with anti-GFP (GTX113617, GeneTex, Irvine, CA) or anti-FLAG (F1804, MilliporeSigma, Saint Louis, MO) antibodies.

## 3 Results

### 3.1 Identification of a novel variant in the *SEPTIN12* gene in infertile men

**FIGURE 1:**
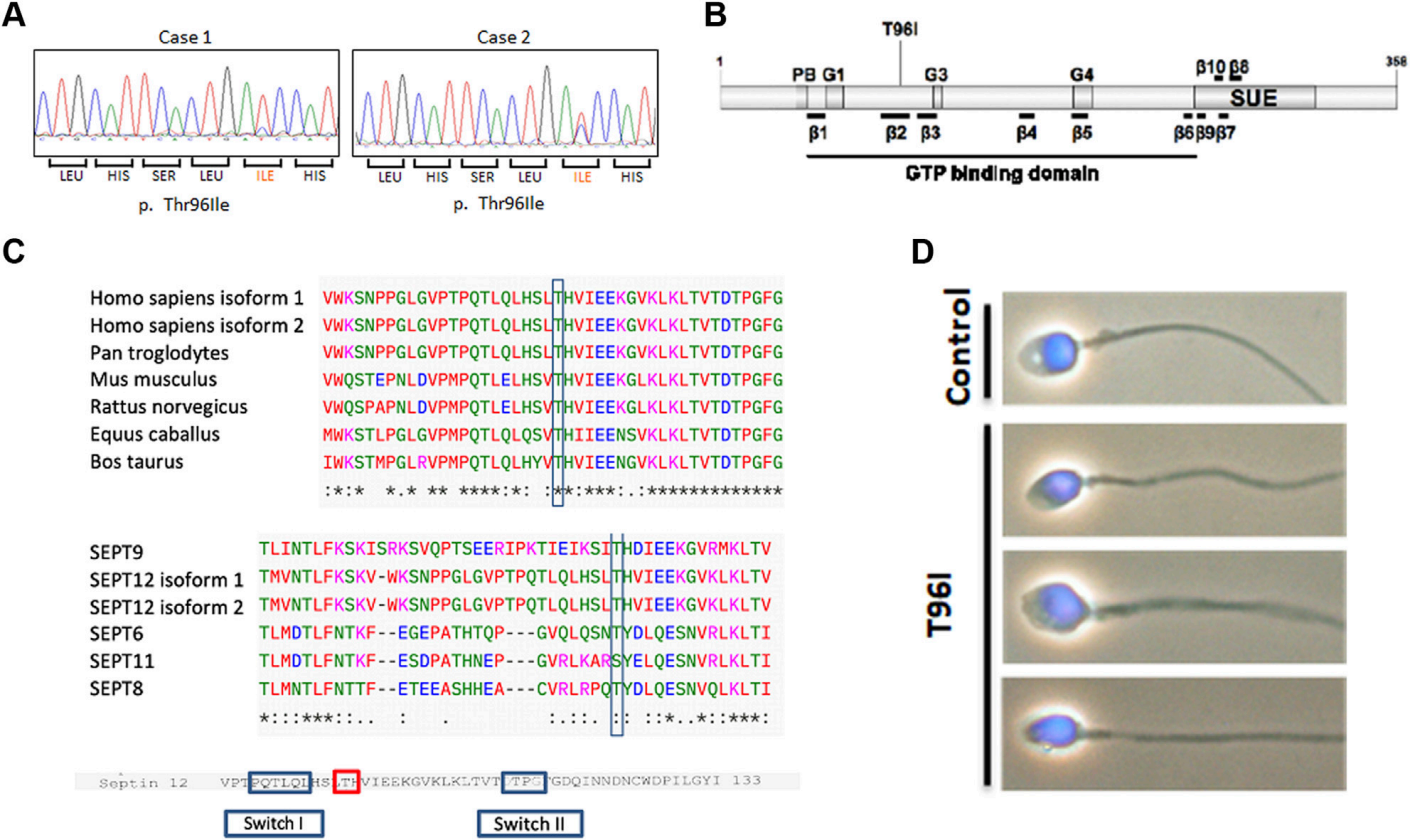
A novel *SEPTIN12* variant is identified in two patients with infertility. **(A)** The panels show the corresponding sequences from the patients with nucleotide changes (c. 287T > C). **(B)** Schematic diagram of the *SEPTIN12* locus, showing novel nonsynonymous nucleotide changes (c. 287T > C) in two infertile men. **(C)** Comparison of amino acid sequences flanking the Thr96 in SEPT12 of different species (up). Comparison of amino acid sequences flanking the Thr96 in human SEPTs (bottom). The amino acid sequences were interpreted using the ClustalW2 program at EMBL-EBI. **(D)** The abnormal sperm morphology in the *SEPTIN12* ^T96I^ patient. The bright-field microscopic images merged with DAPI staining.

Previously, we screened 400 infertile men and 360 fertile men to assess whether the *SEPTIN12* mutation was responsible for male infertility (Kuo et al., 2012). We sequenced the ten exons of *SEPTIN12* by Sanger sequencing and detected novel dominant mutations in exon (c.287T > C) in 2 infertile men (Figure 1A). The mutation is found in β -sheet 2 within the GTPase domain, replacing threonine with isoleucine (T96I) (Figure 1B). The variant is present in the Genome Aggregation Database (gnomAD v4.1.0, http://gnomad.broadinstitute.org/) with an allele frequency of 8.68 × 10^−6^ and was reported as uncertain significance. This missense variant impacts conserved residues in multiple species and the SEPT family (Figure 1C). Two patients, a 40-year-old and a 46-year-old man, were diagnosed with infertility with a normal chromosome karyotype (46; XY) (Table 1). We conducted semen analysis according to World Health Organization (WHO) guidelines and found that patient one had significantly decreased sperm motility (Asthenozoospermia), and patient two had abnormal sperm morphology (Teratozoospermia) (Table 1). Sperm morphology was evaluated using light microscopy, revealing abnormally shaped sperm heads in the patient, including large, small, and thin heads according to WHO guidelines (WHO, 2021) (Figure 1D). These findings suggest that *SEPTIN12* ^T96I^ mutation may be a potential pathogenic factor causing infertility in patients.

**TABLE 1:** Clinical evaluation.

Amino acid variation	Age (years)	Karyotype	Clinical feature	Ratio	Sperm count	Morphology	Total motility
p. Thr96Ile (T96I)	40	46, XY	Asthenozoospermia	Patient: 2/400, Control: 0/360	68 × 10^6^/mL	32% normal 68% abnormal	0%
p. Thr96Ile (T96I)	46	46, XY	Teratozoospermia	Patient: 2/400, Control: 0/360	77 × 10^6^/mL	4% normal 96% abnormal	42%

Ratio: The ratio of cases that harbors mutant allele.

### 3.2 Sperm head and annulus defects in the case carrying *SEPTIN12* ^T96I^

**FIGURE 2:**
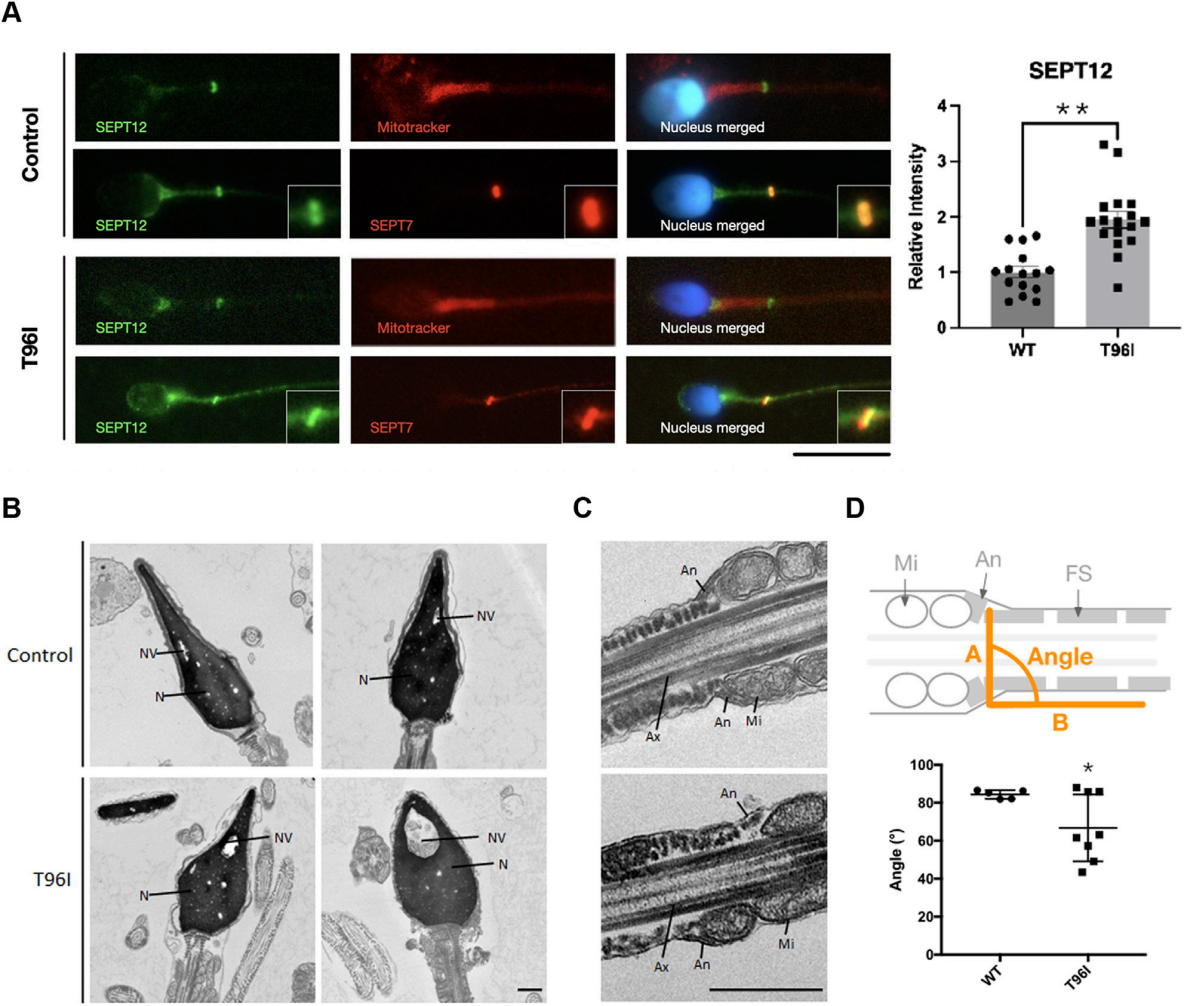
T96I mutation in *SEPTIN12* is linked to head and annulus defects. **(A)** The T96I mutation of spermatozoa was immunostained with anti-SEPT12 antibodies (green), anti-SEPT7 antibodies (red), Mitotracker (red), and DAPI (blue) (left). The fertile man’s sperm as the control. Scale bar, 10 μm. The intensities of SEPT12 in the necks of WT (n = 15) and T96I (n = 17) were quantified using ImageJ (right). **(B, C)** Electron microscopy analysis of WT and *SEPTIN12* T96I mutation of spermatozoa. In WT spermatozoa, the normal acrosome (Ac), nucleus (N), nuclear vacuoles (NV), annulus (An), mitochondria (Mi), and fibrous sheath (FS) were observed. In the patient’s case, the sperm head and annulus location were abnormal. Ax, axoneme. Scale bar, 0.5 μm. **(D)** Schematic representation of mouse spermatozoa (up). The angle between A line and B line. A line is a connection between two annuli. B line along with fibrous sheath. Quantitative representation of the angle between A line and B line (bottom). (Control, N = 5. T96I, N = 8). The data are presented as the means ± SEM. *P < 0.05.

Having identified *SEPTIN12* ^T96I^ in infertile men, we next examined the sperm phenotypes. Mature sperm cells are composed of a head, neck and a tail, and the tail is further divided into a midpiece, principal piece, and end piece (Miyata et al., 2024). The annulus, a SEPT ring, is surrounded by a mitochondrial sheath in the midpiece and a fibrous sheath in the principal piece (Lin et al., 2019). Our previous study showed that SEPT12 was at the annulus (Shen et al., 2017). Immunofluorescence analysis of T96I sperm showed that the SEPT12 signal was located at the annulus and neck. T96I sperm exhibited mitochondrial morphology similar to normal sperm (Figure 2A). Immunofluorescence analysis of T96I sperm showed co-localization of SEPT12 and SEPT7 in the annulus; however, the annulus seemed to be obliquely positioned (Figure 2A). Transmission electron microscopy (TEM) was utilized to examine details of sperm structure. Unlike the normal control, the T96I sperm exhibited large nuclear vacuoles and irregular swelling and decondensation of the acrosomal cap (Figure 2B). The presence of large nuclear vacuoles signifies abnormal chromatin packaging crucial for fertilization. The decondensation of the acrosomal cap in T96I sperm indicated that T96I is associated with nuclear and acrosome defects. Under TEM, the sperm annulus was positioned obliquely, a finding consistent with the immunofluorescence staining (Figure 2C). The abnormal positioning of the annulus in T96I sperm was quantified in Figure 2D. T96I was localized in the neck and showed an increase in expression (Figure 2A), but TEM analysis suggests it was not associated with neck defects. The exact mechanism remains unclear and needs further investigation. This data suggests that *SEPTIN12* ^T96I^ is associated with subtle annulus defects.

### 3.3 Effects of SEPT12 mutation on SEPT12-SEPT7 filament formation

**FIGURE 3:**
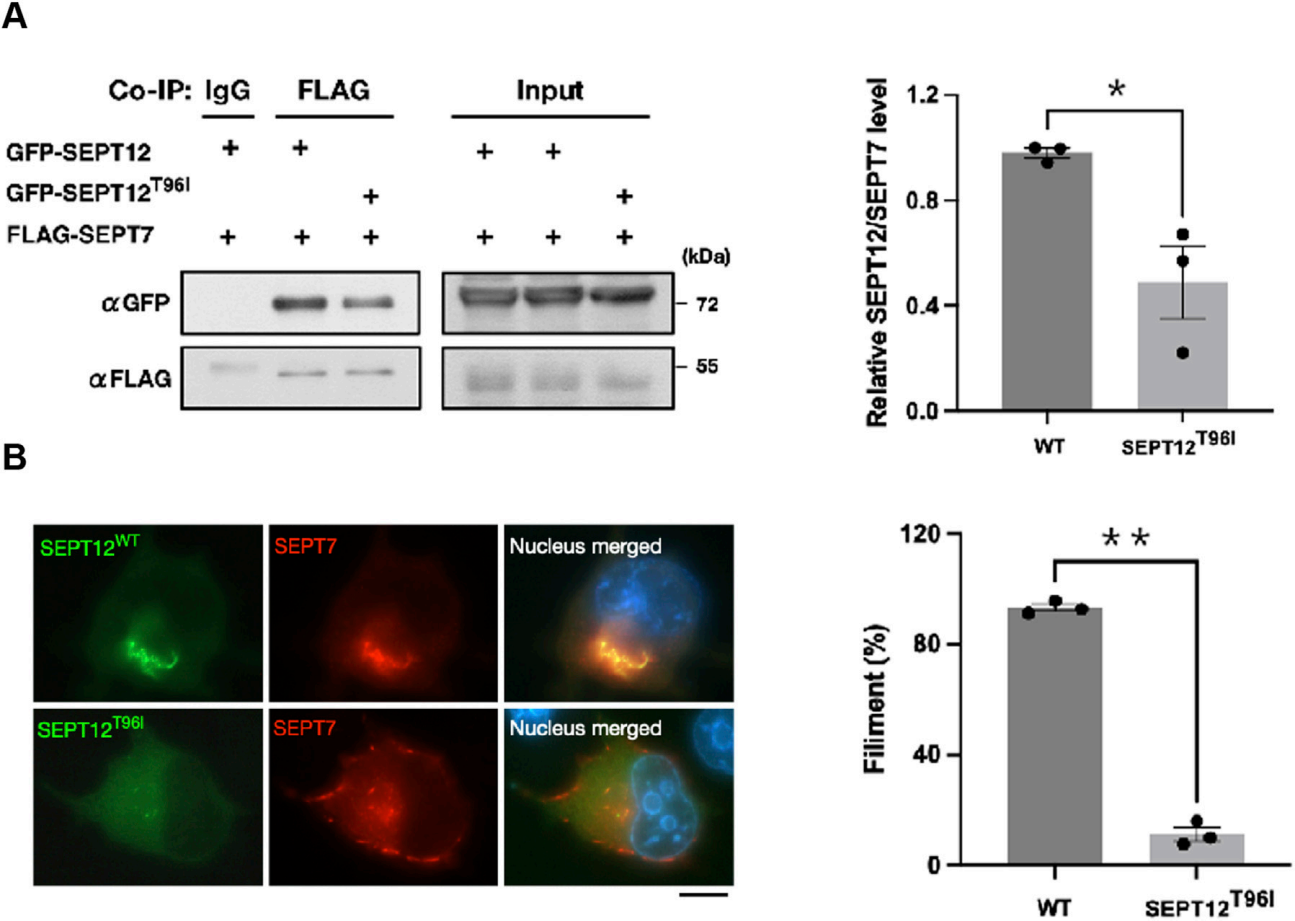
The T96I mutation in SEPT12 disrupts the formation of SEPT12-SEPT7 filaments. **(A)** NT/2D1 cells were transfected with GFP-SEPT12 or GFP-SEPT12^T96^, together with FLAG-SEPT7 as indicated. The cell lysates underwent immunoprecipitation followed by Western blot analysis (left). The band intensities of SEPT12 and SEPT7 were quantified using ImageJ, and their relative ratios are shown in the graph (right). The data are represented as the means ± SEM (n = 3). *P < 0.05. **(B)** NT/2D1 cells were transfected with GFP-SEPT12 or GFP-SEPT12^T96^, together with FLAG-SEPT7. Cells were immunostained with anti-FLAG antibodies (red) and DAPI (right). Scale bar, 10 μm. Filament fibers were quantified by counting SEPT12-SEPT7 fibers in 50 observed cells per experiment (left). The data are represented as the means ± SEM (n = 3). **P < 0.005.

Our previous study indicated that SEPT2 forms an octameric core complex assembled in the order of SEPT2/4-SEPT6-SEPT7- SEPT12–SEPT12–SEPT7–SEPT6–SEPT2/4 in the sperm annulus (Kuo et al., 2015). During spermiogenesis, SEPT12 containing-filaments first appear around the acrosome at step 7 (Lin et al., 2009). By steps 10-11, they form a circular structure between the acrosome and the manchette (Lin et al., 2011). As mitochondria develop, SEPT12 containing-filaments move to the sperm neck and annulus. In mature sperm, they are mainly found in the head, neck, and midpiece, with fewer signals in the tail (Lin et al., 2019). Given switching threonine (hydroxyl, hydrophilic) for isoleucine (aliphatic, hydrophobic) could potentially alter both the structure and function, we hypothesized that T96I mutation affects the assembly process, disrupting SEPT12-SEPT7 filament formation and causes head, acrosome and annulus defects. To explore this concept, we generated a SEPT12 mutant in which T96 residue was changed to I96 for our study. The coimmunoprecipitation results indicate that the T96I mutant exhibits decreased binding to SEPT7 compared to the WT (Figure 3A). Immunofluorescence analyses were used to examine the SEPT12-SEPT7 filament formation in NT2/D1 cells. SEPT7 and SEPT12 WT were co-localized and formed SEPT12-SEPT7 filaments in NT2/D1 cells, but SEPT12 T96I was diffused in the cytoplasm without forming the filaments (Figure 3B). Analysis of cells with SEPT12-SEPT7 filaments showed that the SEPT12 T96I mutation resulted in reduced filament formation (Figure 3B). Our *in vitro* data demonstrate that the SEPT12 T96 residue is important for the SEPT12-SEPT7 filament formation. However, 3D modeling showed that T96 residue is located within the β-sheet of SEPT12 (Supplementary Figure S1) and does not seem to affect the protein folding of SEPT12 or SEPT12/SEPT7 interaction. Interestingly, we observed that the T96 residue is located at a solvent-exposed region. The substitution of threonine (hydroxyl, hydrophilic) by isoleucine (aliphatic, hydrophobic) at this position may impact the stability of the local structure of SEPT12 (Supplementary Figure S2D). Taken together, these data suggest partial disruption of SEPT12/SEPT7 interaction by T96I mutation.

## 4 Discussion

Given expression or SEPT12 in sperm head, neck, midpiece, and annulus during spermiogenesis, pathological variants of *SEPTIN12* are expected to cause qualitative defects of different subcellular compartments (Lin et al., 2019). Previous studies have reported that *SEPTIN12* mutations, including c.266C > T/p; Thr89Met (Kuo et al., 2012), c.616del; p.A206Pfs*10 (Li et al., 2021), c.589G > A/p; D197N (Kuo et al., 2012), E282A (Quarantani et al., 2023), and c.474G > A (Rafaee et al., 2020) cause male infertility. Sperm carrying *SEPTIN12* ^D197N^ variants show impaired structure in the sperm neck and annulus, leading to decreased sperm motility via interference with guanosine-5′-triphosphate (GTP) binding and adversely affecting SEPT 12 filament formation (Kuo et al., 2012). Sperm with *SEPTIN12* ^T89M^ variants and a frameshift mutation (A206Pfs*10) have abnormal sperm heads and a small acrosome (Kuo et al., 2012; Li et al., 2021). SEPTIN12 ^T89M^ reduced GTP hydrolytic activity, adversely affecting SEPT 12 filament formation (Kuo et al., 2012). Additionally, sperm carrying *SEPTIN12* ^E282A^ variants are associated with asthenoteratozoospermia and annulus defects (Quarantani et al., 2023). E282A is located in the GTP-binding domain and has the potential to affect GTP binding. Nevertheless, additional experimental validation is essential to confirm this mechanism. Finally, *SEPTIN12* c.474G > A was associated with a higher risk for sperm head and annulus defects, bent tail, or tail loss (Lin et al., 2012; Rafaee et al., 2020). The c.474G > A mutation resulted in a truncated SEPT12 protein lacking the C-terminal half and part of the GTP binding domain (Lin et al., 2012; Rafaee et al., 2020). The c.474G > A mutation filament formation (Lin et al., 2012). These findings suggest that *SEPTIN12* is a causative gene of head, neck, and annulus defects in humans. In the present report, two patients carried the same genetic variant but were presented with distinctive phenotypes: Asthenozoospermia without obvious nuclear or tail defects in case 1; Teratozoospermia (acrosomal, nuclear, and annulus defects) in case 2. Other factors, including genetic backgrounds or lifestyles, may jointly modify the phenotypic effects of this pathological variant.

The present study identified a novel *SEPTIN12* ^T96I^ variant that causes male infertility. Given the importance of SEPT12 containing filaments for sperm morphology and function, this locus may offer valuable insights for detecting male infertility in the future.

### 4.1 Limitations

Revealing the precise role of SEPT12 T96I in the cytoskeleton and its impact on sperm morphology is essential for advancing our understanding of reproductive biology. First, exploring SEPT12 variants in a larger cohort of patients is essential due to the sample size limitations. This will help clarify the effects of SEPT12 on sperm morphology in infertile men and further confirm the role of the SEPT12 mutation in cases of asthenozoospermia or teratozoospermia in future studies. Further, this may aid in identifying T96I as a biomarker for infertility in the future. Second, due to a lack of sperm cells available from the patient, it is difficult to provide the mechanical mechanisms of how SEPT12 T96I engages with other SEPTINs or cytoskeleton proteins to affect sperm morphology. Two patients with the same T96I mutation show different phenotypes, and the reason remains unclear due to the lack of additional samples. Future studies should use *Sept12* ^T96I^ knock-in mice to deepen our understanding of its mechanistic insights, phenotype (e.g., asthenozoospermia or teratozoospermia), and biological significance.

## Data Availability

The original contributions presented in the study are included in the article/Supplementary Material, further inquiries can be directed to the corresponding author/s.
