# Supplementary material for: A novel SEPT12 mutation, T96I, is associated with sperm head and annulus defects
Source: Front Cell Dev Biol. 2025 Jan 7;12:1498013. doi: 10.3389/fcell.2024.1498013 (PMC11756531; doi:10.3389/fcell.2024.1498013)
Supplement: Supplementary file 1 [file DataSheet1.docx]

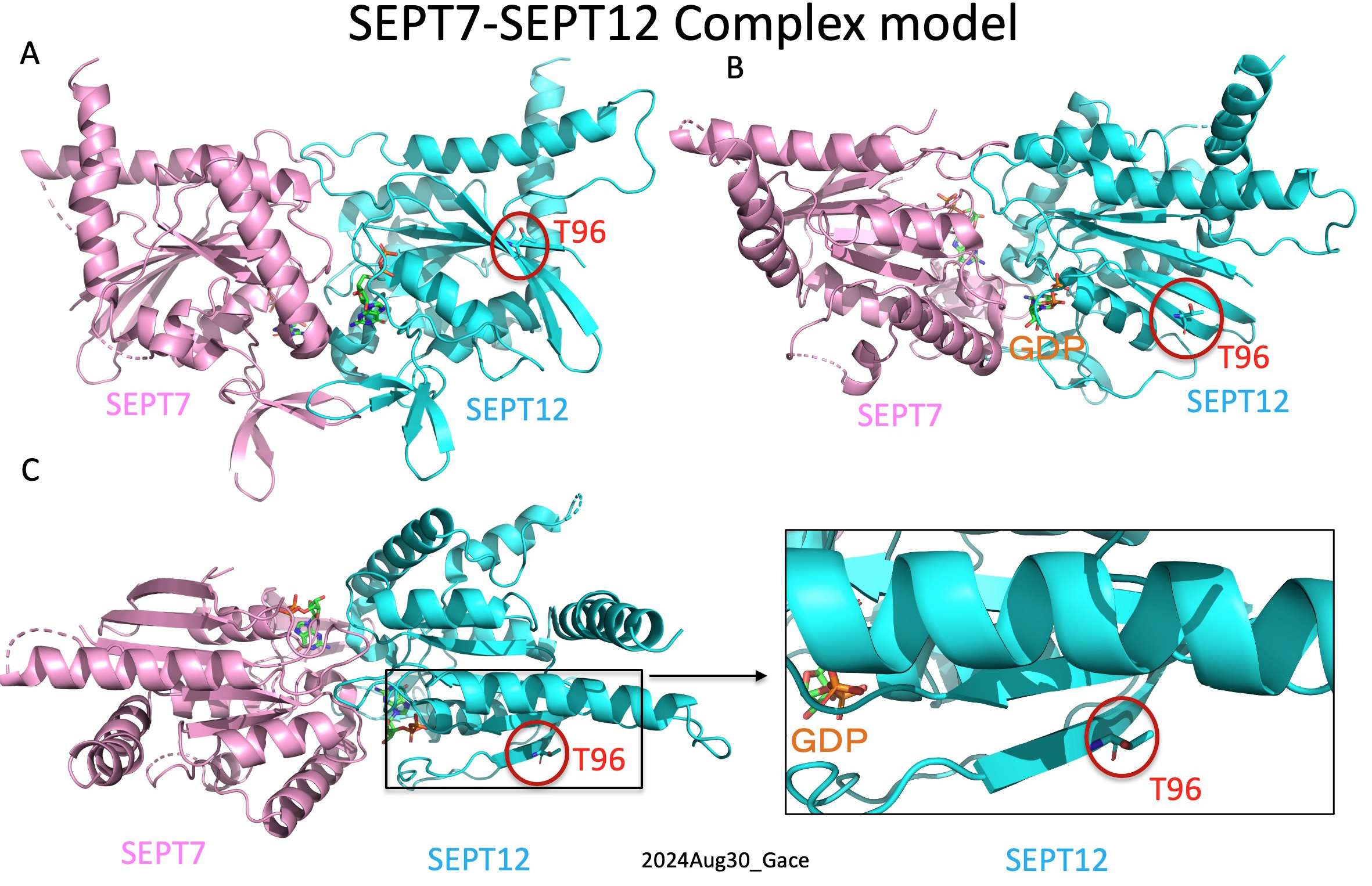


**Supplementary Figure 1 The visualization of the 3D structure of the SEPT7 and SEPT12 (SEPT7-SEPT12) complex model.** A, B, and C represent different orientations, respectively. D. Show an enlarged view of the T96 residue location in SEPT12.
